# The potential impact of the collaborative efforts of physiotherapy, occupational therapy and biokinetics to manage geriatric healthcare

**DOI:** 10.17159/2078-516X/2026/v38i1a27174

**Published:** 2026-10-15

**Authors:** BD Doherty, TJ Ellapen

**Affiliations:** Department of Human Movement and Therapeutic Sciences, Tshwane University of Technology, South Africa

**Keywords:** geriatric, physical functionality, physiotherapy, occupational therapy, biokinetics, multidisciplinary

## Abstract

Geriatric healthcare presents multifaceted challenges which included diminishing physical functionality, prevalence of non-communicable diseases, neuromusculoskeletal pain and cognitive deficits. Collectively these challenges limit the quality of life of the elderly. In South Africa, the professions of physiotherapy, occupational therapy and biokinetics are involved in geriatric healthcare management, but in isolation and not a part of a multidisciplinary physical rehabilitation team (MPRT). Multidisciplinary rehabilitation teams for geriatric patients are well established internationally providing quality healthcare. This is not the case in South Africa. The potential impact of such a MPRT is intriguing. This commentary highlights the need for a MPRT within the South African context and potential role of biokinetics within the MPRT.

The general principle of multidisciplinary rehabilitation for older adults is well established internationally.^[1]^ Integrated geriatric healthcare management mitigates multifaceted age associated challenges such as reduced physical functional capacity and independence, upsurge in non-communicable diseases and cognitive impairments.^[1,2]^ The practice of multidisciplinary rehabilitation is an investment which benefits the patient and society. The International Social Security Association (ISSA) advocates MPRT to reduce the cost hospitalization, length of hospitalization stays and prevent re-admissions.^[2]^ The MPRT encourages geriatric patients to remain independent, minimizing the need for caregiver support.^[2]^ This type of multidisciplinary rehabilitation approach is lacking within the South African configuration. Some of reasons for the lack of multidisciplinary rehabilitation approach are due to the public healthcare policy, current medical funding models and professional protectionism. The South African public healthcare sector does not employ biokineticists and which has encouraged them to practice within the private healthcare sector. A small percentage of South Africans can afford private healthcare. The current medical funding models do impose limitations on the number of biokinetic rehabilitation sessions a patient is entitled to within a year. Healthcare professional protectionism does occur within the South African healthcare system, which limits patient referrals and collaborations. The ideal would be the collaborative management of South African geriatric healthcare by the professions of physiotherapy, occupational therapy, and biokinetics to bridge the gap between acute clinical treatment (physiotherapy), daily functionality (occupational therapy), and long-term vitality (biokinetics) (Fig 1). This specific MPRT covers the entire healthcare continuum from the pathogenic paradigm (treating illness and risk of illness) to salutogenic paradigm (focusing on factors that support human health and well-being).

The distinctive value of this commentary lies within its encouragement of the establishment of such MPRT specifically within the South African configuration, and the particularly the role of biokinetics within the team. Biokinetics provides crucial benefit to a MPRT as the specialist in final-phase functional rehabilitation. Physiotherapists are acute-phase rehabilitation specialists treating acute pain and early mobilization, while biokineticists use scientifically prescribed exercise and physical activity in the strengthening and functional phases.^[3]^ Occupational therapists translate the clinical (physiotherapy) and functional (biokinetics) benefits into real world independence to perform daily activities independently and return work safely.^[4]^ Biokinetics serves as the link from clinical management to the safe return to daily activity.

Biokinetic rehabilitation for geriatric patients varies from the apparent healthy individuals to the management of non-communicable diseases and age-related conditions.^[5]^ Further, biokinetic rehabilitation as shown to improve cognitive functioning among the geriatric population.^[6]^ Structured exercise and physical activity is one of the tenets of physical rehabilitation prescribed and practised by the profession of biokinetics. The amalgamation of the functional physical activity collaboration based on successive benchmarks functional goals of each profession will remove duplication and loss of time and resources. This communication addresses the specific roles of each profession, and the collaborative benefits of a MPRT to better manage geriatric healthcare.

## Roles of each profession

- A physiotherapist will manage acute neuromusculoskeletal pain, joint inflammation, and restore baseline functional mobility after surgeries and/or stroke.^[3]^ Once the elderly person has achieved baseline physical functionality, they are then referred to a biokineticist and occupational therapist for final-phase rehabilitation and daily functionality respectively. However, if the patient develops neuromusculoskeletal pain, and joint inflammation which limits their physical function, the occupational therapist and/or biokineticist must refer to the physiotherapist (Fig 1).
- The biokineticist will begin the final-phase rehabilitation, prescribing clinical-based personalized exercise and physical activity programmes aimed to improve cardiorespiratory endurance, muscle strength and endurance, and postural stability, which can significantly diminish the risk of falls. Both non-communicable diseases and musculoskeletal final-phase rehabilitation can be supervised by a biokineticist concurrently, which will minimize financial burden on the geriatric patient. The value of biokinetic rehabilitation allows the geriatric patient to improve their physical functional status, which will facilitate easier transition to achieve their occupational therapy goals. The improved physical functionality will improve the person’s self-confidence promoting independence.
- The occupational therapist will translate functional improvements in muscle strength, endurance, and proprioception achieved from the biokinetics rehabilitation into real world autonomy.^[4]^ Occupational therapists will evaluate the physical functionality and cognitive abilities of the person, consequently, adapt the home environment, and teach practical methods for daily activities like bathing, dressing, and cooking. If the occupational therapist identifies specific physical functional deficiency, which need increased muscle strength and endurance, stronger cardiorespiratory endurance and/or proprioception they should refer to the biokinetics to address through physical activity and exercise (Fig 1).

## Collaborative benefits

A major challenge in geriatric healthcare management is the elevated rate of hospital re-admissions due to repeated neuromusculoskeletal injuries or poorly managed non-communicable diseases.^[2]^ A MPRT approach significantly reduces the duration of hospital stays and overall medical costs ^[2]^.

It is hypothesized that MPRT approach within the South African context will enhance the functional Independence of the elderly by breaking down barriers to daily living, empowering them to be less dependent on caregivers. It is further hypothesized that the elderly will be exposed to a non-pharmacological pain management strategy through the alternative of targeted therapeutic exercises to reduce their dependence on analgesic medication for pain. These hypotheses need to be empirically validated.

However, the success of this MPRT is dependant on each professions respecting, appreciating of each other, regular communication, and structured referral system. It would be prudent to acknowledge the possible challenges within the MPRT such as practical considerations of the different professions, simultaneous needs across professions, team coordination, access disparities, and funding. However, with strong planning and mutual cooperation some of these challenges can be safely navigated. The challenges of access disparities and medical funding require a twofold approach. The first step involves the inclusion of the profession of biokinetics into public healthcare sector, and second the development of a MPRT of physiotherapists, occupational therapists and biokineticists within the public healthcare sector which will provide the opportunity for South Africans access to this service at a reduced cost.

## Conclusion

The development of a MPRT of physiotherapists, occupational therapists and biokineticists in the South African public healthcare sector has the potential to benefit its geriatric population, which should be encouraged.

## Figures and Tables

**Fig. 1:**
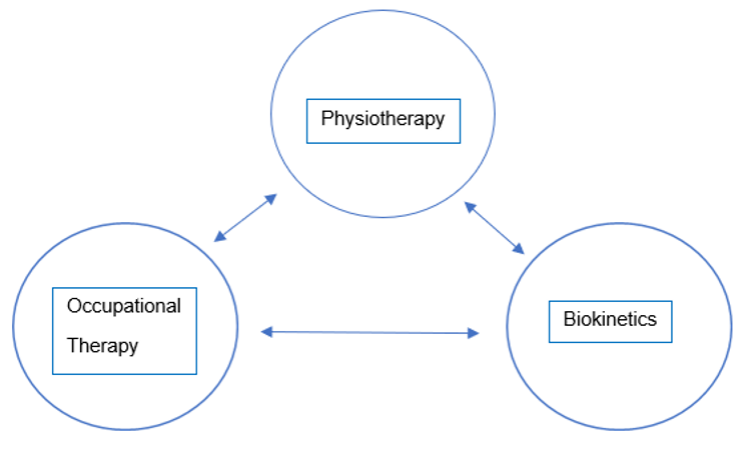
The referral system within the MPRT
